# Editorial: Sustainable human- environment interactions from scientific, technological, and psychological perspectives

**DOI:** 10.3389/fpsyg.2024.1436051

**Published:** 2024-06-07

**Authors:** Tien-Chi Huang, Tai-Kuei Yu, Maria Limniou

**Affiliations:** ^1^Department of Information Management, National Taichung University of Science and Technology, Taichung, Taiwan; ^2^Department of Business Administration, National Quemoy University, Kinmen, Taiwan; ^3^Department of Psychology, Faculty of Health and Life Sciences, University of Liverpool, Liverpool, United Kingdom

**Keywords:** sustainable development, human-environment interactions, psychological perspectives, technological innovations, cultural factors, environmental communication, pro-environmental behavior

The concept of Society 5.0, introduced by Japan in 2016, envisions a future where innovation and technology are harnessed to address societal challenges. This vision emphasizes problem-solving, value creation, interdisciplinary collaboration, resilience, and environmental harmony, aligning with the United Nations' 17 Sustainable Development Goals (SDGs). The United Kingdom's advocacy for “responsible science and technology” further underscores the need for balanced and sustainable approaches to technological advancements. In this context, this Research Topic sought to explore the intricate relationship between sustainable development, human-environment interactions, and the psychological dimensions that shape our responses to these challenges.

After a rigorous review process, these 11 papers were finally selected that fit the theme and made profound research contributions. Other papers that were not accepted, although valuable, were not selected due to various reasons. The Research Topic received 71 submissions, of which 11 were accepted, resulting in an acceptance rate of 15.5%. These accepted papers, representing the contributions of 63 authors, collectively delve into the multifaceted aspects of *Sustainable human-environment interactions from scientific, technological, and psychological perspectives*.

## Sustainable development and human-environment interactions

Within this Research Topic, several papers delve into the interplay between sustainable development and human-environment interactions. Strappini et al. explore how the perception of material properties, affordances, and aesthetics can shape sustainable behaviors, emphasizing the impact of material design on user behavior and sustainability. Zhang L. et al. investigate the influence of collectivist culture on pollution emissions in China, highlighting the importance of cultural factors in implementing environmental policies. Yin et al. utilize a socio-ecological model to understand why Chinese college students participate less in Earth Hour, shedding light on the disconnect between environmental beliefs and actions. Bapoğlu Dümenci et al. assess the impact of renewable energy education programs in museums on children's and parents' awareness of renewable energy and the environment, underlining the role of education in raising awareness. Hou et al. employ prospect theory and evolutionary game models to analyze how government subsidies can promote green supply chains, exploring how policy incentives influence corporate decisions.

These studies collectively emphasize that sustainable development is not solely a matter of technology and policy but is deeply influenced by cultural, psychological, and behavioral factors. Recognizing this interconnectedness is crucial for developing effective strategies to address environmental challenges. By understanding and incorporating cultural values, we can create solutions that resonate with people and foster genuine engagement. Acknowledging individual mindsets and behaviors helps us design interventions that motivate sustainable actions at a personal level. Ultimately, sustainable development is a holistic endeavor that requires us to consider the complex interplay of cultural, psychological, and societal factors to create lasting and meaningful change.

## Technological innovations and sustainability

The role of technological innovations in promoting sustainability is another key focus of this Research Topic. Pol et al. investigate the psychosocial aspects of environmental communication, emphasizing the importance of effective information dissemination in fostering sustainable behaviors. This paper reveals that environmental communication, supported by information and communication technologies (ICT), can significantly influence public attitudes and behaviors toward sustainability. Effective communication strategies that leverage ICT can help mitigate the sense of learned helplessness and enhance public engagement in pro-environmental activities.

He et al. explore the complex relationship between urban built environments and public sentiment, highlighting how improvements in urban design can enhance wellbeing and promote positive emotions toward the environment. Their study underscores the significance of considering both macro and micro environmental factors in urban planning to foster a positive public sentiment and enhance the quality of life in urban settings. By integrating sentiment analysis with urban planning, policymakers can create urban environments that not only meet functional needs but also promote psychological wellbeing. Zhang J. et al. examine the impact of environmental management system certification on green innovation performance. Their research demonstrates that such certifications can significantly enhance corporate sustainability practices by promoting green innovations. The study highlights the importance of corporate social responsibility and the role of environmental certifications in driving green innovation. By adopting environmental management systems, companies can improve their sustainability performance and contribute to broader environmental goals.

## Psychological factors and pro- environmental behavior

Understanding the psychological factors that influence pro-environmental behavior is essential for promoting sustainability. Liang et al. explore residents' preferences for lawns vs. spontaneous groundcovers in urban green spaces, revealing that aesthetic and ecological values significantly influence these preferences. Their findings suggest that promoting spontaneous groundcovers, perceived as more natural and ecologically valuable, can enhance urban biodiversity and sustainability. This research highlights the potential of integrating ecological aesthetics into urban planning to foster public support for sustainable landscaping practices.

Nowakowska and Rönnlund investigate the role of social value orientation and future time perspective in predicting pro-environmental behaviors, suggesting that highlighting future benefits can encourage sustainable practices. Their study shows that individuals with a future-oriented perspective and high social value orientation are more likely to engage in pro-environmental behaviors. This finding suggests that pro-environmental campaigns should emphasize the long-term benefits of sustainable actions to motivate individuals with different value orientations. Neurohr et al. develop a new scale to measure adolescents' interest in nature, finding that higher interest levels correlate with greater engagement in pro-environmental activities. Their research provides a valuable tool for assessing adolescents' interest in nature and suggests that fostering this interest can enhance their engagement in sustainability efforts. By incorporating nature-related activities into educational programs, educators can cultivate a generation that values and actively participates in environmental protection.

## Future directions

The diverse contributions to this Research Topic underscore the complexity and interconnectedness of sustainable human-environment interactions. They highlight the need for interdisciplinary research that bridges the gap between scientific, technological, and psychological perspectives. Future research should continue to explore the psychological factors that influence pro-environmental behaviors, the role of technology in shaping our relationship with the environment, and the development of effective strategies for promoting sustainable development. By integrating insights from diverse fields, we can foster a deeper understanding of the challenges and opportunities that lie ahead in creating a more sustainable and harmonious future for both humanity and the planet.

## Author contributions

T-CH: Data curation, Supervision, Writing – original draft, Writing – review & editing. T-KY: Supervision, Writing – review & editing, Validation. ML: Supervision, Validation, Writing – review & editing.

